# Expression of carbonic anhydrases IX and XII during mouse embryonic development

**DOI:** 10.1186/1471-213X-6-22

**Published:** 2006-05-23

**Authors:** Heini Kallio, Silvia Pastorekova, Jaromir Pastorek, Abdul Waheed, William S Sly, Susanna Mannisto, Markku Heikinheimo, Seppo Parkkila

**Affiliations:** 1 Institute of Medical Technology, University of Tampere and Tampere University Hospital, Biokatu 8, FIN-33520 Tampere, Finland; 2 Center of Molecular Medicine, Institute of Virology, Slovak Academy of Sciences, Bratislava, Slovak Republic; 3 Edward A. Doisy Department of Biochemistry and Molecular Biology, Saint Louis University School of Medicine, St. Louis, Missouri, USA; 4 Children's Hospital and Program for Developmental and Reproductive Biology, University of Helsinki, Helsinki, Finland; 5 Department of Pediatrics, Washington University, St. Louis, Missouri, USA; 6 Department of Clinical Chemistry, University of Oulu, Oulu, Finland

## Abstract

**Background:**

Of the thirteen active carbonic anhydrase (CA) isozymes, CA IX and XII have been linked to carcinogenesis. It has been suggested that these membrane-bound CAs participate in cancer cell invasion, which is facilitated by an acidic tumor cell environment. Since active cell migration is a characteristic feature of embryonic development, we set out to explore whether these isozymes are expressed in mouse embryos of different ages. The studies were focused on organogenesis stage.

**Results:**

Immunohistochemistry demonstrated that both CA IX and XII are present in several tissues of the developing mouse embryo during organogenesis. Staining for CA IX revealed a relatively wide distribution pattern with moderate signals in the brain, lung, pancreas and liver and weak signals in the kidney and stomach. The expression pattern of CA XII in the embryonic tissues was also relatively broad, although the intensity of immunostaining was weak in most tissues. The CA XII-positive tissues included the brain, where the most prominent staining was seen in the choroid plexus, and the stomach, pancreas, liver and kidney.

**Conclusion:**

Membrane-bound CA isozymes IX and XII are expressed in various tissues during mouse organogenesis. These enzymes may regulate ion and pH homeostasis within the developing embryo.

## Background

The carbonic anhydrases (CAs) are a group of zinc-containing metalloenzymes that catalyse the reversible hydration of carbon dioxide in a reaction CO_2 _+ H_2_O ↔ H^+ ^+ HCO_3 _^-^. They are produced in a variety of tissues, where they play important roles in a number of biological processes such as acid-base balance, respiration, carbon dioxide and ion transport, bone resorption, ureagenesis, gluconeogenesis, lipogenesis and body fluid generation [1-3]. Thirteen enzymatically active alpha CAs have been reported in mammals so far, of which CA I, II, III, VII, and XIII are cytoplasmic [4], CA IV, IX, XII, XIV, and XV are anchored to plasma membranes [5-8], CA VA and VB are mitochondrial [9], and CA VI is the only secretory form, present in saliva and milk [10,11].

Of the thirteen active isozymes, CA IX and XII have been linked to neoplastic invasion [12,13]. Both are transmembrane proteins. CA IX is composed of four domains: an N-terminal proteoglycan domain, a CA catalytic domain, a transmembrane region and a short cytoplasmic tail [14]. It is a highly active enzyme, and its activity can be efficiently inhibited by sulfonamides [15-19]. In addition to its enzyme activity and role in pH control, CA IX is a cell adhesion molecule and may also contribute to cell proliferation [20-22]. The distribution of CA IX has been studied in adult human, rat and mouse tissues [5,23]. The most abundant expression of CA IX was observed in the human alimentary tract, particularly in the mucosa of the stomach and gallbladder, and it was also detected in the ileum, colon, liver and pancreas. In mouse tissues, the highest immunoreactivity for CA IX was reported in the gastric mucosa, while moderate signals were also seen in the colon and brain and lower expression in some other tissues, including the pancreas and various segments of the small intestine. CA IX is ectopically expressed at relatively high levels and with a high prevalence in some tumor tissues whose normal counterparts do not contain this protein, e.g. carcinomas of the cervix uteri, esophagus, kidney, lung and breast [24-29]. On the other hand, tumors originating from tissues with high natural CA IX expression, such as the stomach and gallbladder, often lose some or all of their CA IX upon conversion to carcinomas [30-32].

CA XII contains an N-terminal extracellular domain, a putative transmembrane α-helix and a small intracellular C-terminal segment with potential phosphorylation sites [6,14,33]. Its expression has been demonstrated by immunohistochemistry in the adult human kidney, colon, prostate, pancreas, ovary, testis, lung and brain [34,35], and the enzyme has been localized to the basolateral plasma membranes of the epithelial cells [36-38]. In the human kidney, CA XII is confined to the proximal and distal tubules and the principal cells of the collecting duct [39]. In mouse tissues it is most abundant in the kidney [40] and the surface epithelial cells of the colon [41]. CA XII expression also shows a clear association with certain tumors, being overexpressed in renal cancer cells, for example [6].

One characteristic feature of embryonic development is active cell migration from one place to another. Although this clearly represents a benign process, it has some mechanistic similarities to cancer cell invasion [42,43], e.g. the fact that the moving cells invade through the extracellular matrix. Since CA IX and XII probably participate in neoplastic invasion, we set out to explore how these isozymes are expressed during embryonic development.

## Results and discussion

**Figure 1:**
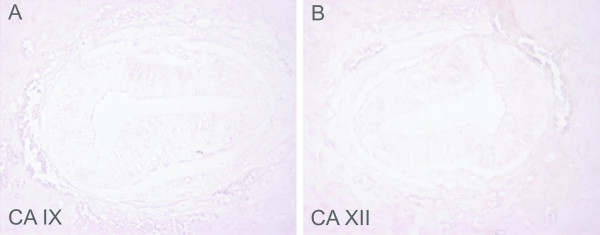
Immunostaining of CA IX and CA XII in the embryos at E7.5. No immunoreaction is detected for either CA IX (A) or CA XII (B). Original magnifications: × 400.

**Figure 2:**
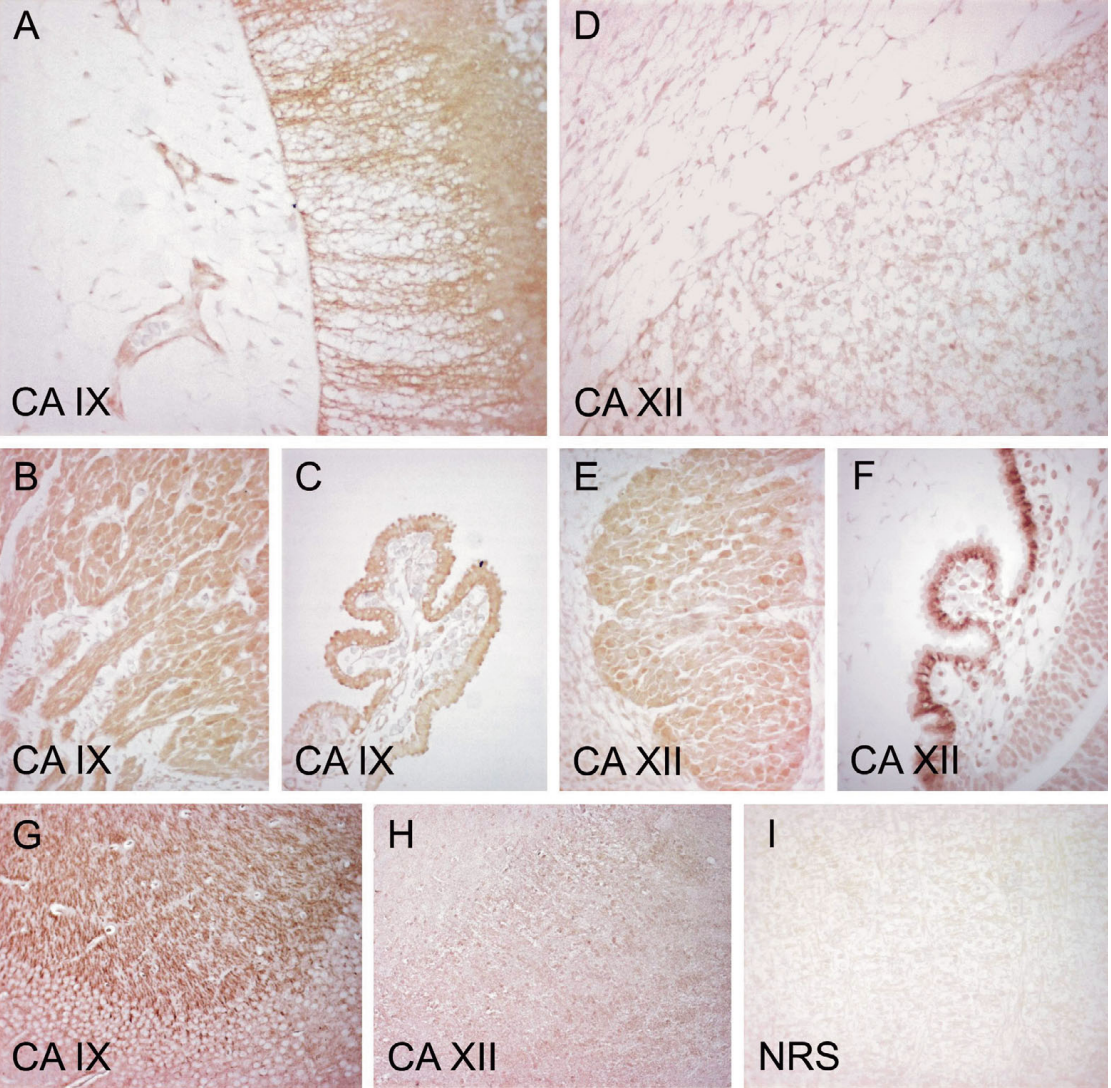
Immunostaining of CA IX and CA XII in embryonic and adult mouse nervous tissues. All embryos are aged E12.5 except the choroid plexus for CA XII, which is aged E13.5. CA IX shows moderate staining in the embryonic brain (A), with the signal mainly located in the neurons. CA IX is also present in the trigeminal ganglion (B) and the choroid plexus (C). Panel G shows strong positive staining for CA IX in the adult brain. CA XII gives weak staining in the embryonic brain (D), but panel E shows moderate staining in the trigeminal ganglion. The strongest immunoreaction is located in the chroid plexus (F). No specific signal for CA XII is detectable in the adult brain (H) except for the choroid plexus (data not shown). Control immunostaining of the embryonic brain with normal rabbit serum is negative (I). Manual PAP staining in panels A-E and I, automated immunostaining in panels F-H. Original magnifications: A-E, I × 400, F × 630, G-H × 200.

**Figure 3:**
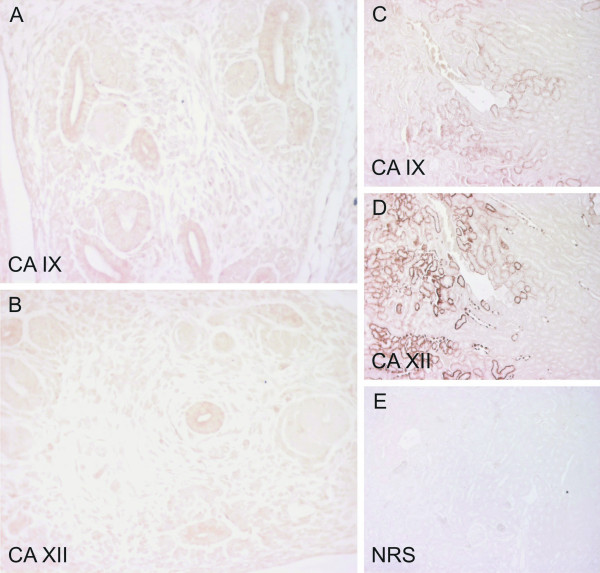
Immunostaining of CA IX and CA XII in the kidney of E12.5 mouse embryos and in the adult mouse kidney. Both CA IX (A) and CA XII (B) show weak staining in the ductal epithelium of the embryonic tissue, and a positive immunoreaction is seen for both isozymes in the adult mouse renal tubules (C, D), with CA XII also located in the collecting ducts. Control immunostaining of an adult mouse kidney with NRS gave no positive signal (E). Manual PAP staining in panels A-B and E, automated immunostaining in panels C-D. Original magnifications: A-B × 400, C-E × 100.

**Figure 4:**
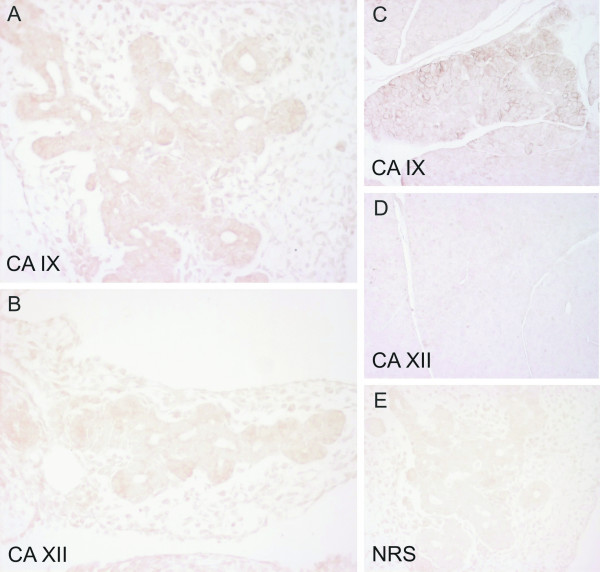
Immunostaining of CA IX and CA XII in the embryonic (E12.5) and adult mouse pancreas. The reaction for CA IX is moderate in the embryonic tissue, with the most intense staining in the epithelial cells (A). CA XII gives weak staining in the epithelium (B). A quite strong but focal signal is seen for CA IX in the acinar cells of the adult pancreas (C), while no immunoreaction is detected for CA XII (D). The control immunostaining of the mouse embryonic pancreas is negative (E). Manual PAP staining in panels A-B and E, automated immunostaining in panels C-D. Original magnifications: A-B, E × 400, C-D × 100.

**Figure 5:**
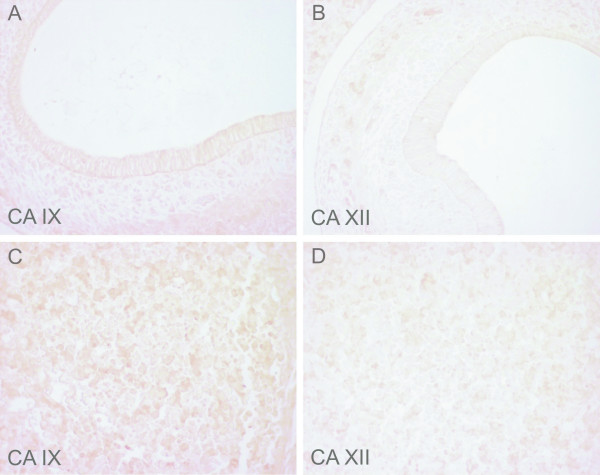
Immunostaining of CA IX and CA XII in the embryonic (E12.5) mouse stomach and liver. Both CA IX (A) and CA XII (B) show weak immunoreaction in the stomach (CA XII barely detectable). CA IX gives moderate staining in the liver, the signal being seen in scattered cells (C). Panel D shows a weak positive signal of CA XII in the liver (D). Manual PAP staining in panels A-D. Original magnifications: × 400.

Immunohistochemical staining of CA IX revealed a relatively wide distribution pattern, although the signal intensity most often remained low or moderate. The E7.5 embryos, representing a gastrulation stage, were completely negative (Figure 1). CA IX expression in the various tissues during organogenesis is summarized in Table 1. The protein was present in the developing brain at all ages studied (Figure 2). The brain tissue was stained moderately, and some positivity was occasionally observed in cells present in the mesenchyme beneath the developing brain (data not shown). Moderate staining was also seen in the nerve ganglia and choroid plexus (Figure 2). No immunoreaction for CA IX was detected in the kidney at E11.5, whereas a weak positive signal appeared at E12.5 (Figure 3). The developing pancreas showed a moderate positive reaction at E12.5, which was primarily seen in the basolateral plasma membrane and intracellular compartment of the epithelial cells (Figure 4). Weak staining for CA IX was present in the stomach at all ages studied (Figure 5). This is in accordance with the finding that CA IX is functionally important for a normal gastric histological structure [44]. The liver also showed positive immunostaining in scattered cells (Figure 5). Positive labeling was seen in the lung and heart, tissues not expressing the protein in the adult mouse (data not shown). It is notable, however, that the adult heart tissue also gave a slight positive signal with the automated immunostaining method, even though it has been previously considered negative for CA IX [23].

**Table 1:** Distribution of CA IX in mouse embryonic tissues of different age.*

**Organ**	**E11.5**	**E12.5**	**E13.5**
Brain	++	++	++
Heart (ventricle/atrium)	+/++	++/++	+/++
Lung	ND	++	+
Kidney	-	+	+
Pancreas	ND	++	ND
Liver	+	++	++
Stomach	+	+	+
Intestine	+	++	+

* Scores in immunohistochemistry: strong reaction (+++), moderate reaction (++), weak reaction (+), no reaction (-), not done (ND).

The expression pattern of CA XII in embryonic tissues was also relatively broad, although the staining intensity was weak in most tissues. The E7.5 embryos showed no immunoreaction (Figure 1). Results at later stages are summarized in Table 2. CA XII protein was expressed in the brain and nerve ganglia at every subsequent age during organogenesis (Figure 2), most prominently in the choroid plexus at E12.5 and E13.5 (Figure 2), i.e. at the time when the developing choroid plexus usually becomes visible. Interestingly, a weak signal for CA XII was detected in several embryonic tissues, including the stomach (Figure 5), pancreas (Figure 4) and liver (Figure 5), which are all negative in adult mice [41]. No staining was detected in the stomach at E11.5, while a weak positive signal appeared there at E12.5. The liver showed weak or moderate staining for CA XII during organogenesis. It is notable that even though CA XII is highly expressed in the adult mouse kidney, the embryonic kidney showed only a weak signal (Figure 3). Weak immunostaining was also seen in the pancreas, where just a few of the developing ducts were positive (Figure 4). In the heart, the staining became stronger during mouse development (data not shown), but as with CA IX, the specificity of CA XII immunostaining is questionable in this particular organ. However, the control stainings using normal rabbit serum instead of the anti-CA IX or anti-CA XII serum gave no positive signals.

**Table 2:** Distribution of CA XII in mouse embryonic tissues of different age.*

**Organ**	**E11.5**	**E12.5**	**E13.5**
Brain	+	+ (CP +++)	++ (CP +++)
Heart (ventricle/atrium)	+/+	++/++	++/++
Lung	ND	+	+
Kidney	ND	+	+
Pancreas	ND	+	+
Liver	+	+	++
Stomach	-	+	+
Intestine	+	+	+

* Scores in immunohistochemistry: strong reaction (+++), moderate reaction (++), weak reaction (+), no reaction (-), choroid plexus (CP), not done (ND).

CA IX and XII are distinct CA isozymes in that they are overexpressed in certain tumors and subjected to regulation by the von Hippel Lindau tumor suppressor protein/hypoxia pathway [35,45]. In developing embryo, the expression patterns of CA IX and CA XII may also be related to the presence of hypoxia, which is considered essential for proper morphogenesis of various tissues [46]. Hypoxia appears important particularly for development of the brain, myocardial vascularization, lung branching morphogenesis, formation of mesoderm and establishment of various progenitor cells [47-49].

The high catalytic activities of CA IX and XII support their role in acidification of the tumor microenvironment, which in turn facilitates the migration of tumor cells through the extracellular matrix [12,13]. The question is whether CA IX and XII also participate in cell migration during embryonic development. Although the present results provide no functional evidence that CA IX or XII is involved in cell migration during embryogenesis, they do indicate that several cell types in the mouse embryo express these isozymes. Interestingly, both isozymes were present though at quite low level in some embryonic tissues whose adult counterparts do not express these particular proteins or the expression is very low. These findings contrast with prior studies on the developmental regulation of CA IV. This isozyme, like many of the cytosolic isozymes, is expressed at much lower levels in most tissues of the embryo than are found in the adult [50,51].

## Conclusion

Membrane-bound CA isozymes IX and XII are expressed in several tissues of developing mouse embryo. As membrane-bound CAs with an extracellular active site, CA IX and XII represent key enzymes in the maintenance of an appropriate pH in the extracellular milieu. Future studies should therefore be focused on exploring how strictly pH homeostasis is regulated in a developing embryo and what are the possible structural or functional consequences if this homeostasis is disrupted.

## Methods

### Antibodies

Polyclonal rabbit antibodies to mouse CA IX and CA XII have been described earlier [40,44]. Non-immune normal rabbit serum (NRS) was used in the control stainings instead of the specific antisera.

### Immunohistochemistry

Mouse embryos were obtained by mating male and female NMRI mice. The procedures were approved by the animal care committees of Helsinki University and Tampere University. Noon on the day on which the copulation plug was found was considered to represent 0.5 days p.c. 7.5 (n = 2), 11.5 (n = 3), 12.5 (n = 4) and 13.5 (n = 2) p.c. embryos with or without extraembryonic tissues were briefly washed with PBS, fixed with 4% paraformaldehyde and embedded in paraffin. Sections were cut at 5–8 μm and placed on SuperFrost^® ^Plus microscope slides (Menzel; Braunschweig, Germany). Tissue samples from the stomach, heart, brain, liver, kidney and pancreas of an adult NMRI mouse were obtained for control purposes. Immunoperoxidase staining was performed using an automated Lab Vision Autostainer 480 (ImmunoVision Technologies Co., Brisbane, CA, USA). As this automated immunostaining method produced some nonspecific labeling of the nuclei in the embryonal tissues, immunostaining was repeated using a less sensitive but more specific peroxidase-antiperoxidase complex method (manual PAP) to confirm the validity of the results.

The automated immunostaining, performed using Power Vision+™ Poly-HRP IHC Kit (ImmunoVision Technologies, Co.) reagents, included the following steps: (a) rinsing in wash buffer; (b) treatment in 3% H_2_O_2 _in ddH_2_O for 5 min and rinsing in wash buffer; (c) blocking with Universal IHC Blocking/Diluent for 30 min and rinsing in wash buffer; (d) incubation with the primary antibody (rabbit anti-mouse CA IX or XII) or NRS diluted 1:2000 in Universal IHC Blocking/Diluent for 30 min; (e) rinsing in wash buffer for 3 × 5 min; (f) incubation in poly-HRP-conjugated anti-rabbit IgG for 30 min and rinsing in wash buffer for 3 × 5 min; (g) incubation in DAB (3,3' -diaminobenzidine tetrahydrochloride) solution (one drop of DAB solution A and one drop of DAB solution B in 1 ml) ddH_2_O for 6 min; (h) rinsing with ddH_2_O ; (i) CuSO_4 _treatment for 5 min to enhance the signal; and (j) rinsing with ddH_2_O. All procedures were carried out at room temperature. The sections were mounted in Entellan Neu (Merck; Darmstadt, Germany) and finally examined and photographed with a Zeiss Axioskop 40 microscope (Carl Zeiss; Göttingen, Germany).

The immunostaining by the PAP method included the following steps: (a) 3% H_2_O_2 _in methanol for 5 min and washing in PBS for 5 min; (b) treatment with undiluted cow colostral whey (Biotop) for 30 min and rinsing in PBS; (c) incubation with the primary antibody (rabbit anti-mouse CA IX or XII) diluted 1:100 in 1% bovine serum albumin (BSA) in PBS for 1 hr and washing in PBS 3 times for 10 min; (d) treatment with undiluted cow colostral whey for 30 min and rinsing in PBS; (e) incubation with the secondary antibody (swine anti-rabbit IgG; DAKO, Glostrup, Denmark) diluted 1:100 in 1% BSA in PBS for 1 hr and washing in PBS 3 times for 10 min; (f) incubation with peroxidase-antiperoxidase complex (PAP-rabbit; DAKO) diluted 1:100 in PBS for 30 min and washing in PBS 4 times for 5 min; and (g) incubation for 2 1/2 min in DAB solution (6 mg 3,3' -diaminobenzidine tetrahydrochloride; Sigma, St Louis, MO) in 10 ml PBS plus 3,3 μl 30% H_2_O_2_. All incubations and washings were carried out at room temperature. The sections were mounted in Entellan Neu (Merck; Darmstadt, Germany) and finally examined and photographed with a Zeiss Axioskop 40 microscope.

## Abbreviations

CA, carbonic anhydrase

## Authors' contributions

All authors participated in the design of the study. HK, SM, MH and SPar collected the tissue samples. HK and SP drafted the manuscript. JP, SPas, AW and WSS produced and characterized the antibodies. HK performed the immunohistochemical staining. HK, MH and SP analyzed the staining results. All authors read, modified and approved the final manuscript.
